# Editorial: From molecules to function: the world of mesencephalic trigeminal nucleus neurons in health and disease

**DOI:** 10.3389/fncel.2026.1884679

**Published:** 2026-06-03

**Authors:** Yong Chul Bae, Hiroki Toyoda, Youngnam Kang

**Affiliations:** 1 Department of Behavioral Sciences, Graduate School of Human Sciences, Osaka University, Suita, Japan; 2 Department of Anatomy, Physiology and Neurobiology, Craniofacial Nerve-Bone Network Research Center, School of Dentistry, Kyungpook National University, Daegu, Republic of Korea; 3 Department of Physiology, School of Dentistry, Aichi Gakuin University, Nagoya, Japan

**Keywords:** Alzheimer-type dementia, central amygdala, mesencephalic trigeminal nucleus, proprioceptive primary sensory neurons, stress

Unlike typical primary sensory neurons, those innervating jaw-closing muscle spindles are located within the brainstem as the mesencephalic trigeminal nucleus (MTN), thereby receiving various synaptic inputs from higher brain regions. While proprioceptive primary sensory neurons typically regulate voluntary isometric contraction, MTN neurons are also involved in malocclusion-induced anxiety and stress, likely due to their diverse synaptic inputs from higher brain regions. Notably, stress often manifests as bruxism, whereas normal mastication aids in coping with stress. Furthermore, chronic malocclusion-induced stress may progress to Alzheimer-type dementia, suggesting an involvement of MTN in its pathogenesis (Kang et al., 2024). It was demonstrated that MTN neurons display two distinct spike waveforms depending on two functional modes: one as primary sensory neurons faithfully relaying spindle impulses to jaw-closing motoneurons (JCMNs), and the other as premotor neurons driving JCMNs independently of spindle activity (Saito et al., 2006). The switching between the two modes is mediated by Nav1.6, GluRs together with mGluR1/5, HCN, and a2A-adrenergic receptors (Chung et al., 2015; Kawasaki et al., 2018; Toyoda et al., 2022). These two modes may provide insights into the intricate involvement of MTN in both malocclusion-induced stress and stress-induced bruxism, which are likely the result of the activation of these modes. This Research Topic aimed to integrate findings on ion channels, receptors, neuromodulators, and transcriptome/proteome data in MTN neurons, which are likely involved in transitioning between the two functional modes of MTN neurons.

In regard to the mode of primary sensory neuron, the review by Lee and Oh provides a comprehensive molecular and developmental framework for MTN neurons, emphasizing their role in coordinated oral motor function and potential links to cognitive

decline. They identified three unique features of MTN neurons that distinguish them from dorsal root ganglion (DRG) proprioceptors: (1) Origin and Location: Unlike neural crest-derived DRG neurons, MTN neurons originate from the dorsal midbrain and reside within the CNS, reflecting their specialized role in complex jaw movements. (2) Molecular Identity: MTN neurons lack Runx3, a transcription factor essential for DRG proprioceptors. Instead, they rely on a distinct transcriptional program, maintaining Ntrk3 and partially Ntrk2 during development. (3) Developmental Refinement: MTN circuits and jaw-closing muscle spindles undergo significant transcriptomic and proteomic remodeling during the transition from sucking to chewing at weaning. Ultimately, they highlight how these embryologically and molecularly distinct neurons are refined to control precise masticatory functions.

Furthermore, Yamada et al. investigate how mesencephalic trigeminal nucleus (NVmes) neurons contribute to chronic jaw muscle pain through activity-dependent neuron–glia interactions. Using an acidic saline-induced pain model, they found that prolonged neuronal hyperexcitability is driven by sustained astrocytic reactivity within the NVmes. A key mediator in this process is the astrocyte-derived protein S100β, which modulates extracellular calcium to facilitate ectopic firing in NVmes neurons. Notably, S100β knockout mice did not exhibit these pain-related neuronal changes, identifying a specific molecular link between glial signaling and sensory dysfunction. The findings demonstrate a causal cascade where maladaptive neuron-astrocyte signaling in a primary proprioceptive nucleus activates downstream nociceptive brainstem circuits. By showing that proprioceptive afferents can actively drive pain signaling, this study challenges traditional sensory boundaries and highlights neuron-glia interactions within primary afferent structures as promising therapeutic targets for persistent muscle pain.

In regard to the premotor neuron mode, Zhao et al. demonstrated the activation of Vme (or MTN) neurons by the central amygdala (CeA), a stress-response center. The overactivity of the masticatory muscles, such as bruxism and teeth clenching, is closely linked to stress and often leads to consistent muscle pain. However, the underlying neural mechanisms have remained unclear. Zhao et al. investigated the pathway between CeA and Vme. Using a chronic stress animal model, researchers found that stress activates GABAergic neurons in the CeA that terminate on GABAergic interneurons within the Vme. While chemogenetic suppression of CeA GABAergic neurons reduced both anxiety and masseter overactivity, specific optogenetic inhibition of the CeA-to-Vme projection reduced jaw muscle hyperactivity without affecting general anxiety levels. These findings indicate that the CeA-Vme GABAergic circuit plays a crucial role in mediating masseter overactivity in response to chronic stress.

Finally, the review by Seki et al. focused on Piezo2 and Nav1.6 to show the involvement of mesencephalic trigeminal nucleus (MTN) neurons in masticatory impairment. MTN neurons regulate masticatory rhythm and reflexes through Nav1.6 channel-dependent ionic mechanisms, which are further modulated by serotonin. However, pathological factors including chronic stress and sodium channel dysfunction can induce MTN hyperexcitability, leading to bruxism and temporomandibular disorders. Additionally, aging and tooth loss cause Piezo2 downregulation, potentially causing masticatory impairment and subsequent cognitive decline. Recent research identifies the targeting of glutamate transporters, melanocortin receptors, and nitric oxide pathways as novel therapeutic strategies. Ultimately, MTNs serve as promising targets for treating a wide range of conditions, from motor disorders to neurodegenerative diseases.
